# Different fundus imaging modalities and technical factors in AI screening for diabetic retinopathy: a review

**DOI:** 10.1186/s40662-020-00182-7

**Published:** 2020-04-14

**Authors:** Gilbert Lim, Valentina Bellemo, Yuchen Xie, Xin Q. Lee, Michelle Y. T. Yip, Daniel S. W. Ting

**Affiliations:** 1 School of Computing, National University of Singapore, Singapore, Singapore; 2 Singapore Eye Research Institute, Singapore National Eye Centre, Singapore, Singapore; 3 Duke-NUS Medical School, National University of Singapore, 11 Third Hospital Road Avenue, Singapore, 168751 Singapore; 4 Vitreo-Retinal Service, Singapore National Eye Center, 11 Third Hospital Road Avenue, Singapore, 168751 Singapore; 5 Artificial Intelligence in Ophthalmology, Singapore Eye Research Institute, 11 Third Hospital Road Avenue, Singapore, 168751 Singapore

**Keywords:** Artificial intelligence, Deep learning, Diabetic retinopathy, Fundus photographs, Retinal imaging modalities, Survey

## Abstract

**Background:**

Effective screening is a desirable method for the early detection and successful treatment for diabetic retinopathy, and fundus photography is currently the dominant medium for retinal imaging due to its convenience and accessibility. Manual screening using fundus photographs has however involved considerable costs for patients, clinicians and national health systems, which has limited its application particularly in less-developed countries. The advent of artificial intelligence, and in particular deep learning techniques, has however raised the possibility of widespread automated screening.

**Main text:**

In this review, we first briefly survey major published advances in retinal analysis using artificial intelligence. We take care to separately describe standard multiple-field fundus photography, and the newer modalities of ultra-wide field photography and smartphone-based photography. Finally, we consider several machine learning concepts that have been particularly relevant to the domain and illustrate their usage with extant works.

**Conclusions:**

In the ophthalmology field, it was demonstrated that deep learning tools for diabetic retinopathy show clinically acceptable diagnostic performance when using colour retinal fundus images. Artificial intelligence models are among the most promising solutions to tackle the burden of diabetic retinopathy management in a comprehensive manner. However, future research is crucial to assess the potential clinical deployment, evaluate the cost-effectiveness of different DL systems in clinical practice and improve clinical acceptance.

## Background

A growing global health problem related to diabetes mellitus, one of the world’s fastest growing chronic diseases, is diabetic retinopathy (DR). This condition has been projected to affect 700 million people across the world within the next two decades [1]. Since one-third of diabetic patients have underlying DR, this would translate to approximately 250 million people suffering from DR by the year 2035 [2–4]. To meet this rapidly evolving and growing crisis, tools that are able to deal with this heavy workload quickly and efficiently are paramount in overcoming and tackling this leading cause of blindness across the world [5, 6].

Early detection of DR via population screening – associated with timely treatment – has been shown to have the potential to prevent visual loss in patients with diabetic retinal complications [7]. Many computer-aided algorithms for automated retina image analysis have been explored [8–12]. Since before the deep learning (DL) era, the development and application of such techniques has produced cost-effective tools for DR screening, [13, 14] and were crucial in the care of patients with DR and other diseases detectable from the retina such as glaucoma, age-related macular degeneration and retinopathy of prematurity [6, 15–17]. Several international research groups have worked on automatic retinal image analysis methods to detect, localize, or measure retinal features and properties, [18–20] such as automated segmentation and diameters measurement of retinal vessels [21].

In this review paper, we present some state-of-the-art DL systems for DR classification using fundus retinal images. We further aim to explain the machine learning (ML) techniques and concepts involved alongside a broad overview of major published works.

### Artificial intelligence in retinal analysis

Artificial Intelligence (AI) is an attractive solution for tackling DR burden. ML is the subfield of AI that focuses on techniques and algorithms that learn to perform tasks without providing specific instructions, and the subset of ML that is DL has garnered particularly huge interest in the last decade [5, 22]. DL was initially inspired by the neuronal connectivity of the brain, allowing it to process large amounts of data and extract meaningful patterns based on past experiences with the same input. Moreover, DL improved on prior and shallower artificial neural networks by being able to model data at various scale abstractions [23]. Specifically, deep convolutional neural networks (CNN) has been at the forefront of this new wave of DL in medical analysis due to its remarkable ability to analyse images and speech with high accuracy. This has resulted in widespread applications in multiple medical specialties, including but not limited to ophthalmology, radiology and pathology [24–28]. CNNs have found particular success in these specialties due to their reliance on imaging data such as fundus photographs, radiological films and pathological slides [24–27].

The validation of such methods is key for demonstrating the robustness and applicability of DL technologies among clinicians, eye care providers, and biomedical scientists [15, 29]. Large and rich sets of testing data are required for the development, as well as comprehensive expert annotations as reference gold standards [30]. To be effective, a high level of confidence in the agreement between the computer system and expert human readers is required. Sensitivity, specificity, accuracy, positive and negative predictive value, and AUC are common statistical analysis to assess the algorithm’s output validity. Also, DL-based systems might serve as a promising solution to reduce human grading workload, and also serve as a cost-effective screening alternative for both high- and low-resource countries [31–33].

Ophthalmology has been at the forefront of this revolution, and DL-based methods are expected to increasingly influence routine clinical patient care in the future [16, 33]. In particular, Abràmoff et al. was the first group to obtain United States (US) Food and Drug Administration (FDA) approval for the use of a DL system in the diagnosis of DR from retinal images [34]. As for Google AI Healthcare, Gulshan et al. demonstrated high diagnostic ability for detecting DR whilst optimizing and minimizing the size of the training dataset required to achieve these results [35]. Ting et al. was able to translate this clinically by demonstrating the high performance of a DL-based system across multi-ethnic populations, despite not originally being trained with eyes of differential phenotypical characteristics, while being subject to non-optimal real-world image capture settings [26]. DL has also found success in detecting other ocular diseases from colour fundus photographs such as age-related macular degeneration, [36] glaucoma [37] and retinopathy of prematurity [38].

Despite many publications attesting to the robustness, reliability and accuracy of these DL systems in the detection of pathological states, and the support garnered from federal agencies such as the US FDA, translation into clinical practice has not been without its challenges [16, 39]. Resistance to implementation has been largely due to the inscrutability of these algorithms [33]. This is due to the ‘black box’ concept that is evident in DL methods describing the ambiguity as to how these networks arrive at their conclusion [5]. Although this is a phrase commonly put forth during the analysis of the applications of DL systems, it holds significant weight in the field of medicine, where accountability for incorrect decisions weigh heavily, and where the patients’ and physicians’ trust is necessary for acceptance of a novel method [16]. That said, there exist methods that are introduced that help to address this issue, including saliency heatmaps that provide a visual representation of regions that DL systems consider in making a decision, or feature attributions where values are assigned to features and those with higher values suggest areas that are critical to the prediction by the model [40–43]. Such methods provide a certain reassurance with DL implementations, and allow for further translational progress.

## Main text

### Retina fundus imaging modalities

**Fig. 1:**
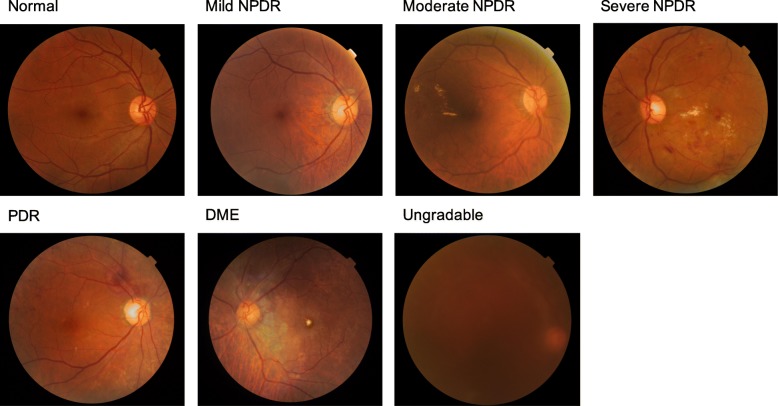
Examples of retinal fundus images

**Fig. 2:**
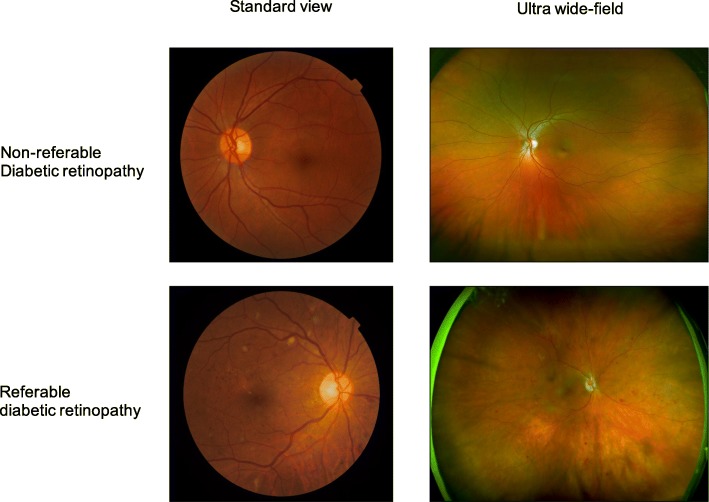
Comparison of standard view and ultra-wide field retinal images with and without referable diabetic retinopathy

Fundus imaging is an established modality for retinal imaging, and the detection of DR from fundus images has a long and rich history in retinal analysis [44]. Fundus imaging is defined as the process whereby reflected light is used to form a two dimensional representation of the three dimensional retina, the semi-transparent, layered tissue lining the interior of the eye projected onto an imaging plane [45]. Figure 1 shows different levels of DR severity from retinal colour fundus images and Fig. 2 provides a comparison of retinal photographs obtained from different types of devices and capturing views. Table 1 summarises the major publications in retinal analysis using DL, separately describing standard multiple-field colour fundus photography, and the newer sub-modalities of ultra-wide field photography and smartphone-based photography. The approaches used for the various studies are also included in the table.

**Table 1:** Summary of the major publications in retinal analysis using DL, grouped by standard multiple-field fundus photography, ultra-wide field photography and smartphone-based photography

Authors and year of publication	Approach	Training dataset	Validation datasets	Performance
Standard view photography				
Gulshan et al. 2016 [35]	Inception-V3 network	Public EyePACS and Messidor-2 (> 120,000 images)	Public EyePACS-1 and Messidor-2 (> 10,000 images)	EyePACS-1
				AUC: 0.99
				Sensitivity: 90%
				Specificity: 98%
				Messidor-2
				AUC: 0.99
				Sensitivity: 87%
				Specificity: 99%
Abràmoff et al. 2016 [46]	AlexNet/VGG network	Public Messidor-2	Public Messidor-2 (~ 2000 images)	AUC: 0.98
				Sensitivity: 97%
				Specificity: 87%
Ting et al. 2017 [26]	VGGNet-19 network	Proprietary SiDRP 2010–2013 (> 76,000 images)	Proprietary SiDRP 14–15 and 10 others (> 112,000 images)	SiDRP 2014–2015
				AUC: 0.93
				Sensitivity: 91%
				Specificity: 92%
				Others
				AUC range: 0.89 to 0.98
				Sensitivity range: 92 to 100%
				Specificity: 76 to 92%
Gargeya et al. 2017 [47]	Customised CNN network	Public EyePACS-1 (> 75,000 images)	Public EyePACS-1, Messidor-2,E-Ophtha (> 17,000 images)	EyePACS-1
				AUC: 0.97
				Sensitivity: 94%
				Specificity: 96%
				Messidor-2 and E-Ophtha
				AUC range: 0.83 to 0.95
				Sensitivity range: 74 to 93%
				Specificity range: 87 to 94%
Abràmoff et al. 2018 [34]	AlexNet/VGGNet network	Public Messidor-2	Proprietary Primary care sites (~ 900 patients)	Sensitivity: 87%
				Specificity: 91%
Keel et al. 2018 [48]	Inception-V3 network	Public LabelMe (~ 59,000)	Proprietary Endocrinology outpatient services (96 patients)	Sensitivity: 92%
				Specificity: 94%
Kanagasingam et al. 2018 [49]	Inception-V3 network	Public and proprietary DiaRetDB1, EyePACS, Australian tele-eye care (30,000 images)	Proprietary Primary care (~ 200 patients)	Sensitivity: 92%
Gulshan et al. 2019 [50]	Inception-v4 network	Public EyePACS and Messidor-2 (> 144,000 images)	Proprietary Two eye hospitals (~ 6000 images)	AUC range: 0.97 to 0.98
				Sensitivity range: 89 to 92%
				Specificity range: 92 to 95%
Raumviboonsuk et al. 2019 [51]	Inception-v4 network	Public EyePACS and Messidor-2 (> 120,000 images)	Proprietary Hospitals and health centers (~ 30,000 images)	AUC: 0.99
				Sensitivity: 96.9%
				Specificity: 95.3%
Bellemo et al. 2019 [52]	VGGNet/ResNet network	Proprietary SiDRP 2010–2013 (> 76,000 images)	Proprietary Mobile screening unit (> 4000 images)	AUC: 0.97
				Sensitivity: 92%
				Specificity: 89%
Ultra-wide field photography				
Wang et al. 2018 [53]	EyeArt software	–	Proprietary Eye clinics (~ 1500 images)	AUC: 0.85
				Sensitivity: 90%
				Specificity: 54%
Nagasawa at al. 2019 [54]	VGGNet-16 network	Proprietary Hospitals (< 400 images)	Proprietary Hospitals (< 400 images)	AUC: 0.97
				Sensitivity: 95%
				Specificity: 97%
Smartphone-based photography				
Rajalakshmi et al. 2018 [55]	EyeArt software	–	Proprietary Tertiary care diabetes hospital (~ 300 images)	Sensitivity: 96%
				Specificity: 80%
Natarajan et al. 2019 [56]	Remidio software Inception-V3 network	Public and proprietary EyePACS and hospitals (> 52,000 images)	Proprietary Population-based screening (> 4000 images)	Sensitivity range: 96 to 100%
				Specificity range: 79 to 88%
Rogers et al. 2019 [57]	Pegasus software	–	Public and proprietary IDRiD and research laboratory study (> 6000 images)	AUC range: 89 to 99%
				Sensitivity range: 82 to 93%
				Specificity range: 82 to 94%

#### Standard view

Standard colour fundus photography provides a 30 to 50-degree image which includes the macula and optic nerve. It is widely used in clinical and trial settings as it provides relatively good documentation of DR. Multiple images can be manually overlapped to create a montage for example, 7 standard 30 degree colour fundus images may be combined to produce a 75 degree horizontal field of view [58]. With the addition of mydriasis, the proportion of ungradable photographs may be reduced from 26 to 5% (*p* < 0.001) [59].

AI systems have generally been shown to be able to accurately detect DR from colour fundus photographs. During the early development and validation of the screening performance of DL systems, most scientific groups evaluated their CNN performances in developed countries, mostly on the United States population [35, 46, 47]. In 2016, Abràmoff et al. developed and enhanced a DL system which achieved a AUC of 0.98 and an achievable sensitivity and specificity of 96.8 and 87.0% in detecting referable DR (defined as moderate non-proliferative DR or worse, including diabetic macular oedema) on a publicly available colour fundus dataset (Messidor-2) [46]. Gulshan et al. also reported promising diagnostic performances of their DL system with an AUC of 0.99, and an achievable sensitivity and specificity of above 96 and 93%, respectively, on two publicly available colour fundus datasets (EyePACS-1 and Messidor-2) [35]. Several other notable studies were conducted in the same year, as awareness of the promising abilities of DL in DR screening aroused the interest of the vision science and medical research communities [60–62].

In 2017, Gargeya and Leng customized a CNN model that achieved an AUC of 0.97 with 94% sensitivity and 98% specificity, on five-fold cross-validation using the EyePACS dataset [47]. They further tested it on two external datasets, achieving AUC scores of 0.94 and 0.95, respectively. Ting et al. then evaluated the performance of their DL system in detecting DR, using colour fundus images collected from a Singaporean national DR screening program, and achieved an AUC of 0.94 with an achievable sensitivity and specificity of 91 and 92% [26]. They further validated the system on 10 additional multi-ethnic multi-cohort multi-settings datasets with diabetes and achieved AUCs ranging from 0.89 to 0.98. Concurrently, interest in DL continued to grow, with many noteworthy studies published [53, 63–68].

In 2018, IDX-DR software utilizing Alex/VGGNet features was validated with an external dataset [69] that was also approved for use by the US FDA, [34] having reported a sensitivity of 91% and specificity of 87% in a real-world clinical setting. Other pilot studies have also shown the applicability of such technologies in real-world settings and primary care [48, 49, 70].

There has thus been much sustained interest regarding the application of DL systems for DR. [71–76] The most notable research direction in 2019 was arguably towards assessing the transferability of AI to other less-explored settings, particularly in developing countries. The Google AI group extended their works to Thailand and India. Ruamviboonsuk et al. reported promising sensitivity and specificity of 97 and 96%, respectively, (AUC of 0.99) in a national screening program from local hospitals and health in Thailand [51]. In India, their DL system achieved a sensitivity and specificity of 89 and 92%, respectively, (AUC of 0.96) on data from the Aravind Eye Hospital, and 92 and 95%, respectively, (AUC of 0.98) on data from Sankara Nethralaya [50]. Bellemo et al. reported a promising sensitivity and specificity (92 and 89%, respectively, with AUC of 0.97) for diagnosis in Zambia, a low middle-income African country [52]. In all the above developing countries, the DL systems’ performance was either superior or comparable to that of human graders. This might provide an impetus for other countries of similar income levels to adopt DL systems for their routine national DR screening programmes [75].

Another notable trend has been the use of a DL system as an assistive tool for human graders. Sayres et al. investigated the use of heat maps generated by a DL system as a guidance system for human graders, which led to a significant improvement in diagnostic accuracy as compared to unassisted humans [77]. Keel et al. investigated a method to visualize the areas where their DL system focused in diagnosing DR. [78] Other applications concern the prediction of cardiovascular risk factors from colour fundus images, as well as the estimation of DR prevalence [79, 80]. In addition, a promising field that might be explored is the use of DL for the generation of synthetic retinal images to overcome legal concerns and low disease prevalence [81].

#### Ultra-wide field

Ultra-wide field imaging allows examination of not only the central retinal area but also the peripheral zones, for up to a 200-degree view of the retina [82]; more than 80% of the total retinal surface can be captured in a single image. With its wide coverage, ultra-wide field imaging is able to detect predominantly peripheral lesions in eyes with DR, with more than 50% of the graded lesions present outside the seven standard Early Treatment Diabetic Retinopathy Study fields [83, 84]. The presence and increasing extent of predominantly peripheral lesions have been associated with an increased risk of DR progression. Therefore, the automated analysis of ultra-wide field images could be of value in DR screening, given the prognostic importance of peripheral lesions in predicting the progression to advanced disease [84].

In 2017, Levenkova et al. developed an algorithm for the automatic recognition of DR features, including bright (cotton wool spots and exudates) and dark lesions (microaneurysms and blot, dot and flame haemorrhages) in ultra-wide field images [85]. The algorithm extracted DR features from grayscale and colour-composite UWF images, including intensity, histogram-of-gradient and local binary patterns. The best AUCs for bright and dark lesions are 94 and 95%, respectively, achieved by a Support Vector Machine classifier. Wang et al. also evaluated performance of an automated AI algorithm for detecting referable DR, with 92%/90% sensitivity with 50%/54% specificity achieved for detecting referral-warranted retinopathy at the patient and eye levels, respectively [53]. More recently in 2019, Nagasawa et al. used ultra-wide field fundus images to detect treatment-naïve proliferative DR. Utilizing 378 photographic images to train the DL model, a high AUC of 0.97 with promising sensitivity of 94.7% and specificity of 97.2% was achieved [54].

#### Smartphone-based

Even though fundus cameras are commonly used in developed regions for DR screening, due to the high cost of equipment and lack of adequate number of trained ophthalmic technicians, deployment in rural areas with medically underserved patient populations remains limited [86]. In recent years, several solutions incorporating additional lens elements to smartphone cameras have been developed to provide affordable solutions and scalable approaches to widespread care.

In 2013, Prasanna et al. developed a smartphone-based decision support system attached to a handheld ophthalmoscope, for screening DR using sophisticated image analysis and ML techniques. It achieved an average sensitivity of 86% [87]. After a preliminary study [88], Rajalakshmi et al. assessed the role of an AI system for detection of DR and sight-threatening DR by colour fundus photography taken using smartphone-based retinal imaging system in 2018, and validated it against grading by ophthalmologists [55]. The AI system achieved 96% sensitivity and 80% specificity in detecting any DR, and 99% sensitivity and 80% specificity in detecting sight-threatening DR with a kappa agreement of 0.78 and 0.75, respectively. In 2019, Wei et al. presented a real-time implementation of CNNs as a smartphone app to provide a low-cost alternative to fundus cameras equipped with lenses [89]. Natarajan et al. also evaluated the performance of another offline, smartphone-based AI system, for the detection of referable DR by using the images taken by the same smartphone-based retinal imaging system on different patient groups [56]. The sensitivity and specificity in diagnosing referable DR were 100 and 88%, respectively, and in diagnosing any DR were 85 and 92%, respectively, compared with ophthalmologist grading. Finally, Rogers et al. evaluated the performance of an AI system from images captured by a handheld portable fundus camera collected during a real-world clinical practice. Validation on the detection of proliferative DR resulted in an AUC of 0.92, with an AUC of 0.90 for referable DR. [57]

### Machine Learning Techniques & Concepts

State-of-the-art DL systems for DR classification generally may be understood in terms of the ML techniques and concepts involved. In particular, contributions by different groups may be analysed according to the choices made pertaining to each technique/concept. Here, we provide a broad overview of common techniques/concepts, and the trade-offs and considerations involved.

#### Model architecture

The DL model architecture is a major design choice, as the evidence on natural images strongly suggests that the model architecture used affects the classification performance level that may be attained, on the same training and validation data [35]. There has been constant innovation in terms of general-purpose end-to-end deep network architectures in recent years [90], with some notable examples being LeNet, AlexNet, VGGNet, Inception, ResNet, DenseNet and SENet, roughly in chronological order of publication (Table 2).

**Table 2:** Major deep learning model architecture families and characteristics. Note that there may be multiple variants (usually with different number of layers/parameters) within each architecture family

Architecture family	Original year	Parameters	Layers	Module organization	Example application(s)
AlexNet	2012	~ 60 million	8	Convolutional, Max Pooling	Abràmoff et al. [34], Quellec et al. [66]
VGGNet	2014	~ 180 million	19	Convolutional, Max Pooling	Abràmoff et al. [34], Quellec et al. [66], Ting et al. [26], Gargeya et al. [47], Bellemo et al. [52]
GoogLeNet (also Inception v1)	2015	~ 7 million	22	Inception, Pool+Concat	Takahashi et al. [63]
Inception (v3)	2015	~ 24 million	42	Inception, Pool+Concat	Gulshan et al. [35], Krause et al. [30]
ResNet	2016	~ 60 million	152	Convolutional, Skip Connections	Bellemo et al. [52]
Inception-ResNet (v2)	2016	~ 56 million	164	Residual Inception	–
SqueezeNet	2016	~ 1.2 million(before pruning)	14	1 × 1 Convolutional, Squeeze & Expand Layers	–
ResNeXt	2017	~ 25 million	50	Convolutional (Grouped)	–
DenseNet	2017	~ 20 million	201	Dense, Transition	–

However, for the medical imaging domain in particular, the declared performance of these architectures on large-scale natural image classification may not always be the most relevant, due to other considerations. For one, the relatively small quantity of medical image data available may lead to overtraining and/or difficulties with training to convergence, with more-sophisticated and higher-capacity models. As such, other than the careful application of transfer learning (covered later), older and simpler architectures may sometimes be favoured for particular applications. For example, the VGGNet architecture remains exceptionally suited for the extraction of intermediate features [91], while requiring relatively more weight parameters than other popular architectures [90].

Moreover, end-to-end classification is not the only paradigm for DL in DR screening. For instance, a hybrid approach would be to deploy DL models as low-level detectors that directly target various classes of lesions. Lim et al. trained models similar to LeNet on spatially-transformed representations of candidate lesions proposed by a maximally-stable extremal region detector, [10] while Abràmoff et al.’s IDx-DR X2.1 used models inspired by AlexNet and VGGNet [46]. In these cases, the projected number and location of true lesions can either be directly matched against clinical reference standards, or the detector output vectors may be used as the input to a fusion algorithm that perfoms the final image-level classification.

Another notable consideration for model architectures would be the amount of computing resources required, which is relevant for deployment on consumer devices such as smartphones, embedded systems, and on possibly less-powerful hardware in under-resourced regions. In general, the fewer the number of weight parameters involved in the model architecture, the quicker the inference, ceteris paribus. If the inference time is sufficiently quick, real-time analysis further becomes possible [92]. To this end, lightweight model architectures such as MobileNet [93] and ShuffleNet [94] have been designed for devices with limited computing power. Alternatively, model compression through pruning and parameter quantization may be done [95]. Given the medical implications of DR screening, however, any such trade-offs of performance for speed may need to be carefully considered.

#### Ensembling

Ensembling involves the combination of multiple independent ML classifier models, to produce a final classifier model that generally performs better than any of its constituent models. With DL models, ensembling is commonly and easily implemented by training multiple models – not necessarily of the same network architecture or inputs – separately, and then combining the outputs of these models during inference. Although regularization techniques such as dropout may be utilized during model training as an approximation to ensembling [96], models trained in this way nonetheless yield further performance gains when ensembled, in practice.

The number of models involved in the final ensemble is a trade-off between training/inference time and performance. Generally, the larger the number of independent models used, the better the performance, but with diminishing returns. For example, Gulshan et al. used an ensemble of ten Inception-v3 models [35], Ting et al. used an ensemble of two VGGNet-based models, although with differently pre-processed inputs [26], which was further extended with a ResNet model in Bellemo et al. [52]

Various methods have been employed for integrating the individual model outputs within an ensemble. Perhaps the most straightforward would be to take a linear average over these predictions, as was done for Gulshan et al. [35] and Ting et al. [26] More complex possibilities would include weighted ensembles [25] and the training of a further classifier model over the ensemble output values.

#### Transfer learning

**Fig. 3:**
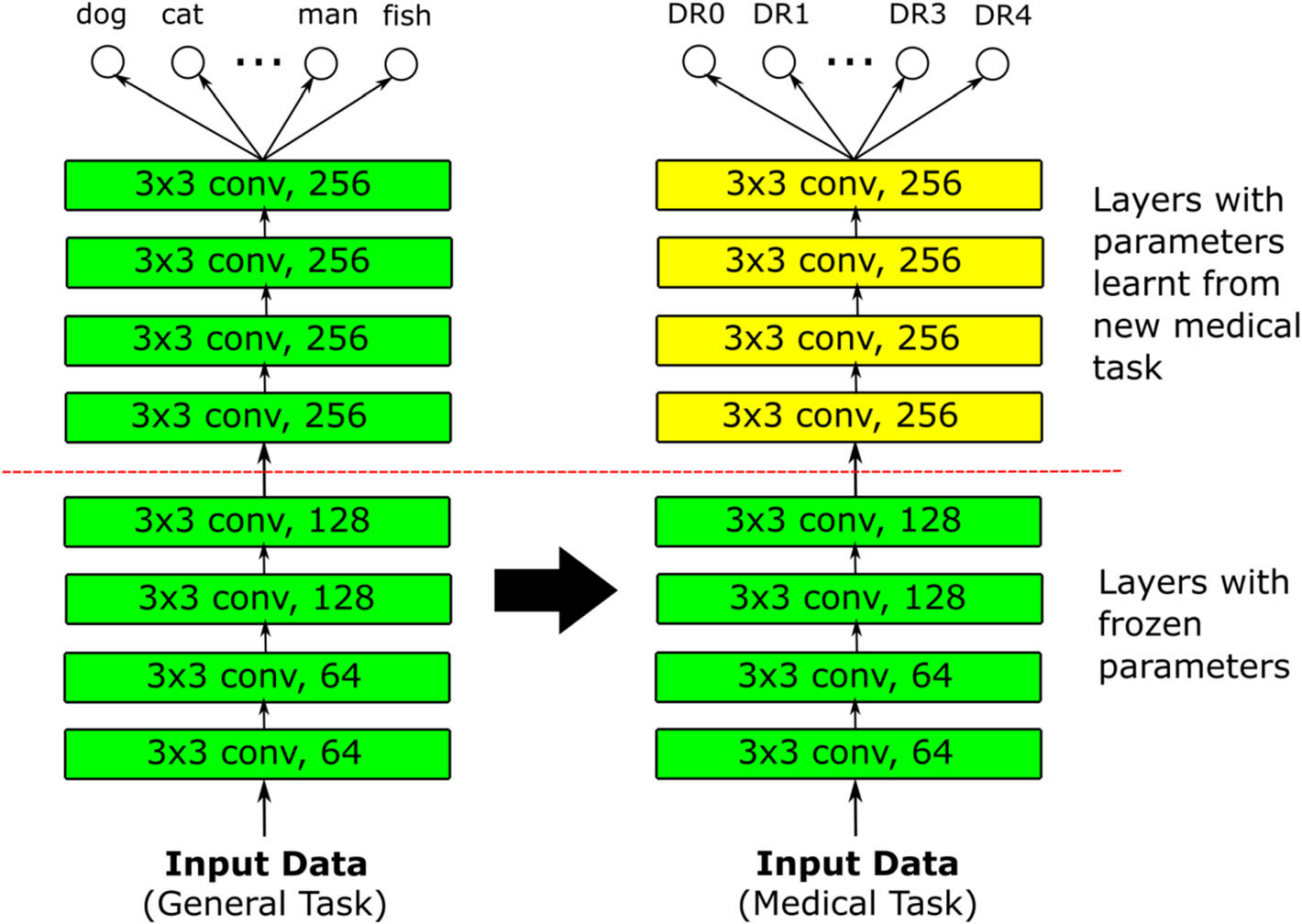
Basic transfer learning method. A deep learning model is first trained on some general task. This trained model is then trained on the actual target medical task, possibly with the parameters for earlier layers representing low-level features frozen

Transfer learning is a method of adapting a model trained on some domain, to another domain (Fig. 3) [97]. For DL models in DR screening, the most prominent application of transfer learning has perhaps been in the finetuning of models that have already been pretrained on another classification task, such as ImageNet [98]. The reasoning behind such transfer learning is that the retinal image domain and the natural image domain share some similarities, especially for universal lower-level features such as corners and edges. Therefore, the parameter weights from a natural image classification task should then serve as a good initialization for retinal image classification.

A major consideration for transfer learning with pretrained weights would be the policy by which these pretrained weights are finetuned with new retinal data. One possible choice would be to consider the pretrained weights merely as an initialization and proceed with training as per normal, allowing all weight values to be updated. At the other extreme, all pretrained weights are fixed, and the pretrained model is effectively employed as a feature extractor with only the output layer replaced, possibly by another classifier such as a random forest [47] or support vector machine [99]. Otherwise, the weights of any number of layers within the model architecture may be fixed, with the remainder updated; if so, it is generally the layers corresponding to lower-level features that are fixed. A previous survey on transfer learning in the medical domain by Tajbakhsh et al. suggests that although the use of pretrained weights made DL models more robust to the size of training sets, the optimal selection of layers to fix depends on the task at hand and has to be empirically determined [98].

#### Weakly supervised and active learning

A commonly encountered obstacle to training DL models for DR classification is a lack of annotated image data, particularly at the lesion level, since such detailed annotation was not typically required in clinical screening workflows. This made gathering sufficient lesion-level ground truth for hybrid DL implementations challenging. Although coarse-grained image-level grades were more widely available, it remained common to have large quantities of unlabelled retinal images for which no grades from human experts were available [100].

In such situations, weakly-supervised transductive learning becomes applicable. In transductive learning, an initial model trained on the labelled training data is used to classify the unlabelled training data. The originally-unlabelled training data now also becomes labelled, and may be used together with the originally-labelled training data to train an improved bootstrapped model [101].

Whether or not such transductive learning is employed, it is advisable to continually refine the trained model through active learning. Active learning presumes the presence of an oracle that can provide accurate answers to queries, which in the case of DR screening would be a human expert. However, there is an opportunity cost to consulting the oracle. As such, the goal of active learning is to intelligently select the most useful images for which to consult the oracle on, in the sense that the availability of accurate labels for these images would improve model performance to the greatest extent. One possible approach would be to select images for which the model is most uncertain [75].

#### Label Modelling

Another manifestation of weakly-supervised learning is the presence of imperfect or noisy labels. The presence of such imperfect labels is largely unavoidable in DR screening, with qualified human graders sometimes disagreeing with each other – or even themselves, from a previous session. Inter-grader kappa scores typically range from 0.40 to 0.65 in DR grading [102], and the implied disagreement may be resolved by majority decision, discussion between the graders, or external adjudication. Krause et al. conclude that rigorous adjudication of DR ground truth is important in developing DR models, since it allows for the principled correction of subtle errors from image artefacts and missed microaneurysms [30].

A further development by Guan et al. has been the modelling of individual graders with independent DL models, following the observation that the labelling of large DR datasets usually involves a large number of human graders, each of whom however grade only a relatively small subset of the dataset, with each image moreover also being graded by only a small subset of the human graders [102]. They found that modelling each human grader separately and averaging the predictions of these separate DL models in a weighted ensemble produced better performance than modelling the expected prediction of the average grader.

#### Joint Learning

DR may co-occur with other related eye diseases, and there is as such motivation to model its features together with those of other eye diseases. This joint or multitask learning involves training a DL model for multiple tasks simultaneously, and may induce beneficial regularization of intermediate representations, thus reducing overfitting [103]. González-Gonzalo et al. attempted the joint learning of referable DR and age-related macular degeneration, and concluded that a jointly-trained DL model could perform comparably to human graders [104].

Joint learning may also be implemented for improving mid-level representations, in terms of optimizing for visual encodings and the final binary classifier at the same time, for multiple-instance learning [105]. This multiple-instance learning framework also allows for a degree of model interpretability by allowing the class of encoding instances to be explicitly considered during training. In this case, two neural networks are utilized to generate the mid-level representation encodings.

#### Hyperparameter search & optimization

Other than the model weight parameters themselves, DL models involve a large number of hyperparameters, such as the initial learning rate, the learning rate decay schedule, the input batch size, etc. For DR screening applications, these hyperparameter settings are often borrowed directly from existing models, and whether these settings are the most appropriate for the DR screening domain may not be systematically explored. Sahlsten et al. is an example of work that investigates the image resolution parameter in detail [106].

The optimization of multiple hyperparameters is non-trivial, due to the number of hyperparameter combinations increasing exponentially with the number of individual hyperparameters. Although grid search over the hyperparameter space is commonly attempted, when the number of relevant hyperparameters is relatively small, random search [107] and sequential optimization algorithms [108] may also be attempted to more thoroughly examine possible model performance.

#### Robustness

Although DL models may be trained and validated on large datasets, it is difficult to be certain whether the datasets used can fully capture the potential variability of retinal images that may be encountered in future use. Differences may arise in the image acquisition process or population demographics that can render a trained DL model less effective on new data. Lim et al. demonstrated that the uncertainty of a DL model could be estimated by the standard deviation and entropy of the mean predictive distribution, on the stochastic batch normalization layers of a ResNet architecture, and that prediction error is correlated with high estimated uncertainty [75].

#### Explainability

A persistent obstacle against the uptake of AI systems in DR screening has been a lack of surface explainability [16]. In fact, the progression from handcrafted features and multi-stage classification to end-to-end deep learning has been accompanied by a concurrent loss of interpretability, in that humans could no longer examine the reasoning of the classifier, unlike previously where an image kernel could be inspected to determine why it had not matched with a microaneurysm, for instance.

**Fig. 4:**
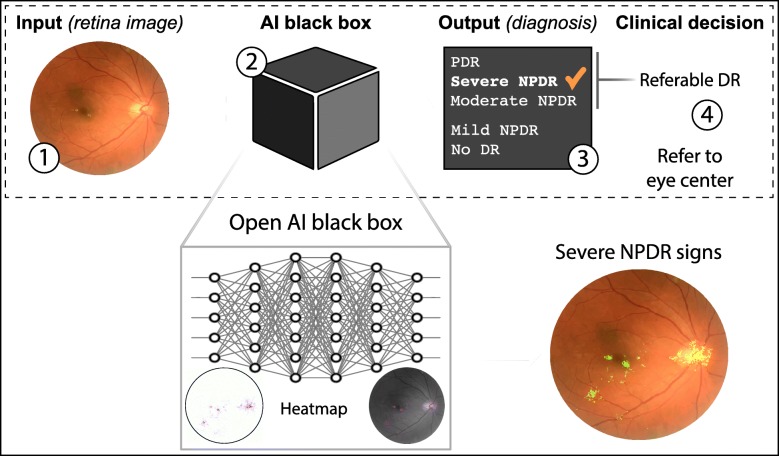
AI flow for diabetic retinopathy. In the diabetic retinopathy screening domain, the AI implementation allows automated diagnosis and subsequent clinical decisions. In the example presented in the figure, the AI system would recommend referring the patient to the eye clinic because of the referable diagnosis for diabetic retinopathy. To allow researchers and clinicians determine how the AI model makes the decision, the heatmap attempts to display the contribution of each image pixel or region, to the final classification. Heatmaps open the ‘black box’ highlighting the areas in which the AI system is focusing on to build trust among practitioners and patients. Abbreviations: DR; diabetic retinopathy; NPDR: non-proliferative diabetic retinopathy; PDR: proliferative diabetic retinopathy

This lack of interpretability has been mitigated somewhat through the development of various methods to extract saliency heatmaps from DL models, such as Grad-CAM [42] and integrated gradients [43]. These saliency heatmaps attempt to display the contribution of each image pixel or region to the final classification. This allows researchers to retrospectively determine whether their DL models are making their decisions based on the expected image features, which in the DR screening domain would be various lesions such as microaneurysms, haemorrhages and hard exudates (Fig. 4).

A desire for greater interpretability has also seen renewed interest in hybrid methods that expose the intermediate goals of the classifier [109]. For example, Yang et al. implemented a two-stage DL model, which first classifies overlapping grid patches as containing lesions or not. The resulting weighted lesion map is then used as input to a second global DL model, to predict the image-level DR severity [110]. Wang et al. introduced a Zoom-in-Net architecture that purports to mimic the attentional behaviour of human graders, by allowing for suspicious regions to be focused on through additional learning on feature maps from the main network [111].

## Conclusions

In this paper, we provided a broad overview of the major works and technical implementations involving DL techniques for DR diagnosis as an alternative tool for screening programmes. It emerged that, in the ophthalmology field, DL tools for DR show clinically acceptable diagnostic performance when using colour retinal fundus images. DL-based AI models are among the most promising solutions to tackle the burden of DR management in a comprehensive manner. However, future research is crucial to assess the potential clinical deployment, evaluate the cost-effectiveness of different DL systems in the clinical practice and improve clinical acceptance.

## Data Availability

Not applicable.

## Abbreviations

DR: Diabetic retinopathy
DL: Deep learning
AI: Artificial intelligence
CNN: Convolutional neural network
AUC: Area under the receiver operating characteristic curve
